# Formation, Application, and Significance of Chicken Primordial Germ Cells: A Review

**DOI:** 10.3390/ani13061096

**Published:** 2023-03-20

**Authors:** Mathan, Gul Zaib, Kai Jin, Qisheng Zuo, Maham Habib, Yani Zhang, Bichun Li

**Affiliations:** 1 Key Laboratory of Animal Breeding Reproduction and Molecular Design for Jiangsu Province, College of Animal Science and Technology, Yangzhou University, Yangzhou 225009, China; 2 Joint International Research Laboratory of Agriculture and Agri-Product Safety of Ministry of Education of China, Yangzhou University, Yangzhou 225009, China; 3 Institute of Epigenetics and Epigenomics, College of Animal Science and Technology, Yangzhou University, Yangzhou 225009, China; 4 College of Veterinary Medicine, Yangzhou University, Yangzhou 225009, China; 5 College of Biotechnology, Jiangsu University of Science and Technology, Zhenjiang 212100, China; 6 Faculty of Biosciences and Agro-Food and Environmental Technology, Royal Veterinary College, University of London, Camden Campus (NW1 0TU) 4 Royal College Street, London NW1 0TU, UK

**Keywords:** primordial germ cell, transgenesis, cryopreservation, gametogenesis, embryogenesis

## Abstract

**Simple Summary:**

The present review illustrates the importance of chicken primordial germ cells to understanding early-stage development for better and more fertile species. By studying early-stage development, we can develop transgenic species to meet food and pharmaceutical demands. This review sheds light on the significance of chicken primordial germ cells and their uses in transgenesis.

**Abstract:**

Chicken is one of the most widely consumed sources of protein globally. Primordial germ cells (PGCs) are the precursors for ova and sperm. One of the early embryogenesis events in most animals is the segregation of the somatic and germ lineages. PGC cultures occur in the germline, and PGCs are less studied in many species. It is relatively challenging to separate, cultivate, and genetically alter chicken without mutating the basic germline. The present study aims to gather previous research about chicken PGCs and provide a customized review of studies and developments in the field of PGCs, especially for avian species. Furthermore, we show that the propagation of chicken PGCs into embryonic germ cells that contribute to somatic tissues may be produced in vitro. Primordial germ cells offer an ideal system in developmental biology, as these cells play a vital role in the genetic modification and treatment of infertility. Cryopreservation helps to maintain genetic resources and sustainable production in the poultry industry. Keeping in mind the significance of cryopreservation for storage and gametogenesis, we discuss its role in the preservation of primordial germ cells. Transgenesis and genetic modifications in chicken lead to the development of various medicinal chicken varieties and aid in improving their production and quality for consumption purposes. Additionally, these characteristics open up new possibilities for modifying the chicken genome for agricultural and medical purposes.

## 1. Introduction

Biopharming has been gaining much more attention in the last few decades. Biopharming is the production and use of transgenic animals and plants to produce pharmaceutical substances for use in animals and humans [1]. Advancements in biopharming have laid a foundation to produce phenotypes such as genetically resistant chickens to meet the global protein consumption demand. Moreover, biopharming offers an excellent method to improve the quality of agriculture and livestock products [2]. Primordial germ cells (PGCs) are promising genetic resources for avian studies, including modified animals. Germ cells are known to transmit information to the upcoming generation with the help of gametogenesis. Gametogenesis is further described in this review. During development, PGCs are the first germ cell population. They are established and are the main origin of both oocytes and spermatogonia [3]. Chicken PGCs are model systems to understand avian and other animals’ gene functions [4]. Previous data have highlighted that PGC migration in all species has similar pathways/mechanisms. Lipids and G-protein-coupled receptors play an essential role in the regulation of PGCs [5]. PGCs are the homogeneous ancestors of gametes. These cells are responsible for transforming genetic data from one cohort to another [6]. In contrast to mammalian transgenic and genome-editing systems, transgenesis and genome editing in chickens and other birds only depend on a special system called germline transmission, involving PGCs. Animal phenotypes, genotypes, and traits are now easily modifiable owing to improvements in precise genome-editing technologies and genetic modification tools [7]. Animal breeders have historically used specific or unnatural breeding techniques to enhance the quality of food, productivity, and many other traits of offspring through the careful breeding of exceptional parents [8]. This method of discriminatory breeding is in line with the outcomes of contemporary genetic engineering or genome editing in terms of the intended genomic sequence of the animal’s DNA. Therefore, it has become possible to create more efficient traits with greater precision [9]. 

**Figure 1:**
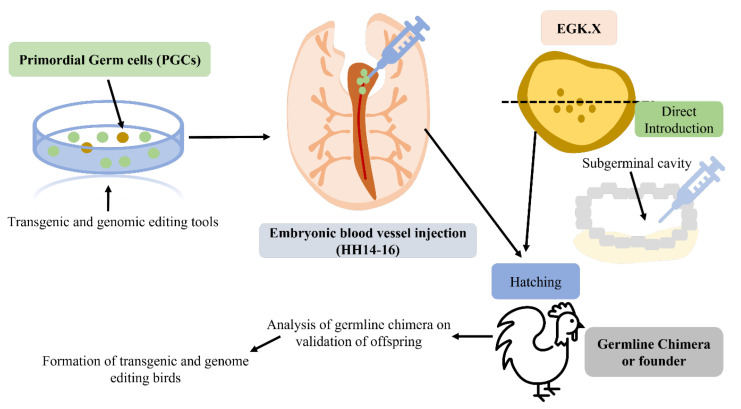
The intrusion of genetically modified PGCs via injection, entering the blood vessels of birds to produce transgenic (TG) and genome-edited (GE) offspring (modified from Han and Park [9]).

In some recent studies, the first step is applying genome modification technology to any selected animal, which invariably entails altering the animal’s germline, allowing the transmission of altered genetic features to the next generations [10]. Figure 1 depicts the production of transgenic (TG) and genome-edited (GE) offspring through the microinjection method. These germline modification techniques are different in different species. The pronucleus of a fertilized mature egg was microinjected with a DNA fragment to produce the first genetically modified mouse in a mammalian species [11]. Additionally, transgenic changes were first used to successfully produce rabbits, lambs, and pigs [12]. This technology is an essential technique in animal transgenesis. Furthermore, recipient genomes randomly contain foreign DNA due to the method’s limited efficacy in producing founder animals. The second major method used for transgenesis in mammals—especially mice [13]—is the use of skilled germline cells such as embryonic stem cells (ESCs). However, birds have a unique system of genetic modification and transgenesis. This is due to the ovum’s physiological characteristics and oviparity [14]. It was found that with a considerable amount of yolk and a short germinal disc, the avian zygote exhibits discoidal meroblastic cleavage, making it challenging to microinject avian ESCs into the blastoderm [15]. Chickens are widely used for transgenesis, as transgenic chickens are disease-resistant, and large-scale production of therapeutic proteins is also achieved. Transgenic chickens have been considered bioreactors for the large-scale production of expensive pharmaceutical proteins. These recombinant proteins are produced in transgenic chicken eggs [16]. Furthermore, recombinant vaccines can also be produced from transgenic chickens. This also provides models for developmental biology research in various fields—especially biotechnology [17]. Transgenesis is a process in which exogenous DNA is introduced into the animal genome. The process involves two steps: knock-in and knockout of a gene. The basic outline of the transgenesis process involves the insertion of the gene of interest into the pronucleus of the fertilized egg right before fertilization, or in the germinal vesicle nucleus. There are various methods for the transfer of genes. Most of the time, the transgenes are submitted through the germline to the ensuing generations [18]. A chicken was the first transgenic avian creature. Many strategies have been produced for the transgenesis of chickens and other birds, including microinjections of transgenes into fertilized eggs [19], subgerminal cavity injections, viral infection of X-stage embryos, and embryonic stem cells [20,21]. However, these methods were unsuccessful in producing genetically altered birds through recombining homologous DNA, due to the low efficiency of germline transmission [22].

To overcome these limitations, primordial germ cells (PGCs) have received a lot of attention as a potential alternative to mammalian germline-competent ESCs [9]. Our main focus in this study is to investigate previous research on chicken PGCs and to provide a customized review, especially for avian species. Additionally, we mention various methods of producing transgenic chickens using PGCs based on gene transfer methods, the basic overview and origin of PGCs, and the derivation and in vitro culture of PGCs, along with their cryopreservation, epigenesis, migration, and propagation.

### 1.1. An Overview of Avian PGCs

**Figure 2:**
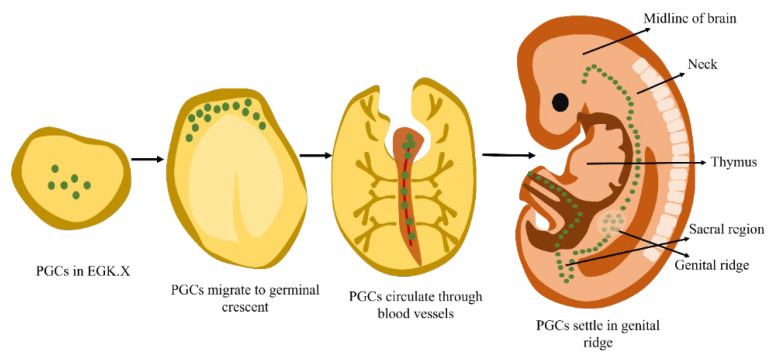
Chicken PGCs are found in the zona pellucida region’s core, migrating via the vascular system and germinal crescent before settling in the genital ridge, which is also the site of the bird’s eventual gonads (modified from Mozdziak and Petitte [29]).

In chickens’ germinal epithelium, germ cells were first observed by Waldeyer in the late 19th century [23]. After this discovery, various other scientists started thoroughly studying germ cells and their origin. It was eventually discovered that PGCs in birds originate from the endodermal region known as the germ wall, which is why they have been given the moniker “germ cells”. Avian PGCs were found in the hypoblast and epiblast layers of the zona pellucida (Figure 2) of EGK stage X blastoderm [9,24]. During the early stage of chicken embryogenesis, until Hamburger–Hamilton (HH) stage 4, PGCs primarily migrate from the zona pellucida’s core region to the germinal crescent region. The glycogen granule content of PGCs is substantial. In order to identify PGCs in chicken embryos, periodic acid–Schiff (PAS) staining is typically utilized. Later, Eyal-Giladi et al. [25] proposed that PGCs arose from the epiblast around EGK stage X based on the results of PAS staining [26]. 

Moreover, avian germline specification is characterized by different variables, which pass down from mothers after finding the chicken VASA homolog (CVH) gene and after the gene’s transcription factors are examined through all developmental stages of the oocyte. This strongly implies the germplasm paradigm of specification is followed by avian PGCs [27]. Additionally, a recent study on the deletion of the azoospermia-like (DAZL) gene in chicken embryos at the intrauterine stage highlights the need for a model of germplasm for avian PGC specification and genesis [28]. 

#### 1.1.1. Origin of Chicken PGCs

In 1976, Eyal-Giladi and Kochav discovered the presence of PGCs in the form of a cluster in the blastoderm of chickens from EGK stage X to HH stage 2 [20]. The subsequent research used chick–quail chimeras, which were studied further and reported in 1981 [25]. Later studies revealed that primordial germ cells emerge in the epiblast layer, and that only the vital part of the blastoderm may increase PGCs [30]. After making this discovery, researchers started looking for more solid evidence for the formation of PGCs. In 1988, Urven et al. examined the establishment of the germline in the embryo of a chicken using an anti-mouse embryonal cancerous cell antibody [31]. They discovered that the monoclonal antibody EMA-1 targets murine EC (Nulli, SCC1) cells and chick primordial germ cells (PGCs). Scientists observed that distinct EMA-1-labeled cells seem to split from the luminal surface of the epiblast and penetrate the blastocoel during the creation of the hypoblast. This occurred concurrently with the generation of morphologically distinct PGCs in the same region—these findings are consistent with the germline’s epiblast origin [31]. Numerous studies supported the same phenomena, and early studies on PGCs are mentioned in Table 1.

Hence, in 2000, scientists studied the germ cell marker chicken vasa homolog (CVH) to distinguish the developmental stages of chicken PGCs. However, investigations on a *Drosophila* vasa homolog in chickens revealed an RNA-binding protein with a germline-specific nature, further demonstrating that the VASA protein in chickens primarily resides in the mitochondrial cloud of oocytes and only confines during cleavage phases in the cleavage furrows [27]. Despite the need for further functional studies, these observations and conclusions imply that the theory of preformation may be the process for determining germ cells in chickens. Functional suppression of germplasm and ectopic transplantation may aid in identifying the mechanisms of avian germ cell specification in the future [3].

#### 1.1.2. Migration of PGCs

The migration of PGCs is a fundamental phenomenon investigated in various species, including avian species, mice, and *Drosophila*. Several genes have been identified for correct germ cell migration. The classic model for PGC migration in *Drosophila*, Tre1 (trapped in endoderm-1), signals through GTPase Rho1 and controls the beginning of migration. The role of migration in chickens is still not developed properly [40]. Much research is still needed to develop the role of migration. In chickens, PGCs go through many stages of proliferation and motility. At EGK stage X, they first remain attached to the hypoblast spreading from the epiblast in the central region of the zona pellucida. Further investigation shows that PGCs first migrate passively, governed or managed by simple streak creation. This movement primarily occurs from the middle region of the zona pellucida to the anterior region. After that, PGCs adhere to basement bands, which are fibrous to the membranes of the zona pellucida. A replacement study using PGC cell lines and DF1 was conducted in 2015. Kang et al. [41] showed that PGCs gradually enter the frontal zone and are actively absorbed into the germline crescent.

**Figure 3:**
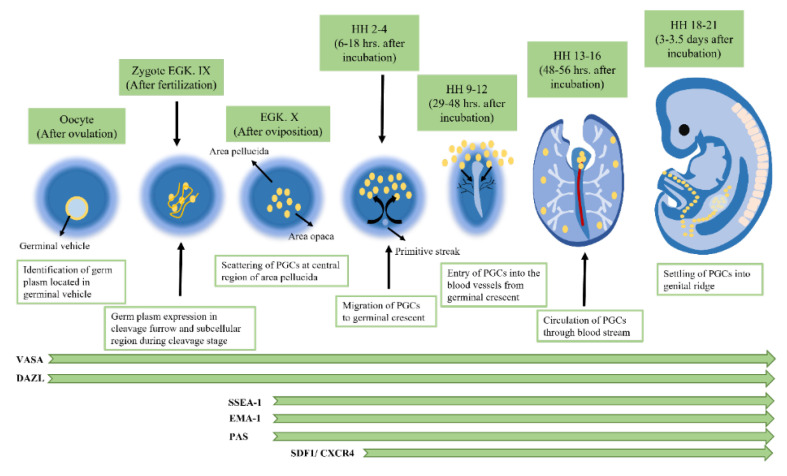
Development of primordial germ cells (PGCs) and expression of markers, shown schematically (modified from Kim and Han [42]).

However, less is understood about the active migration of the germinal crescent, which is regulated by attractive and repellent stimuli. Numerous research findings have shown that some migrating and cultured PGCs produce pseudopodia. This indicates that amoeboid mobility might represent a migration method. PGCs enter the arteries between HH stages 9 and 10, reaching their peak abundance at HH stage 12. At HH stages 15–18, blood-circulating PGCs infiltrate the thicker coelomic epithelium at the genital ridge [42]. Figure 3 illustrates the development of primordial germ cells (PGCs) and the expression of markers.

The migration of PGCs is compatible with changes in various body structures and shapes. In particular, structural alterations in the embryo, which are accompanied by the migration of PGCs to the gonads, essentially permit the transmission of various morphological alterations to the following generation.

### 1.2. Research Progress on PGC Gene Markers and Identification Methods

#### 1.2.1. Derivation of PGCs Using an Indirect Co-Culture Method

**Figure 4:**
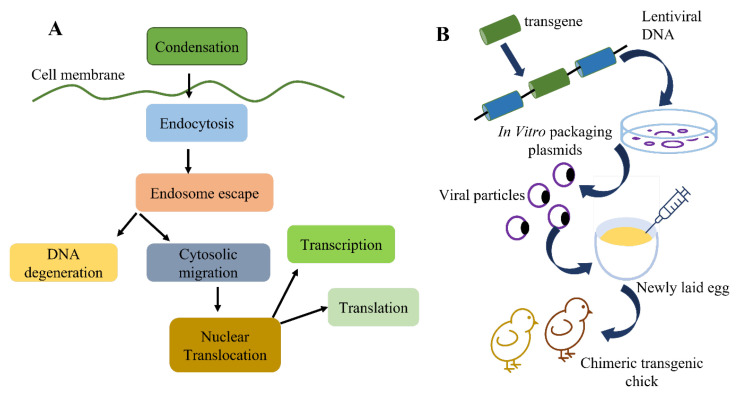
(**A**) Non-viral vector method of transgenesis; (**B**) viral vector transfer of genes (modified from Mozdziak and Petitte [29]).

Avian PGCs are propelled by chemokine receptors during the early stages of embryonic development. They migrate to the growing gonads via the vascular system (Figure 4), which enables both in vitro genetic alteration and isolation for expansion. Previous studies have demonstrated that PGCs separated from the germline crescent, embryonic bloodstream, or embryonic gonads can be transplanted to create germline chimeras [4,43]. This is a promising tool for developing transgenic chickens or preserving genetic resources in endangered birds or domestic avian species. However, it is ineffective, and only a small quantity of PGCs can be recovered from embryonic tissues. Conditional knockout (cKO), Dulbecco’s modified Eagle’s medium (DMEM), and co-culture with feeder layers—such as STO murine fibroblasts, buffalo rat liver (BRL) cells, and chicken embryonic fibroblasts—have been used in several culture systems to increase the production of PGCs (CEF) [44,45].

Adding ovalbumin to the culture medium has also enabled the production of serum- and feeder-free cultures. However, chicken PGCs produced in these culture systems have a poor rate of multiplication and frequently stop growing or start to decline even after a few days in culture. It takes 4 to 8 weeks to develop a cell line (>106 magnitudes) that proliferates from cells adapting to the culture environment. The active proliferation of PGCs in chickens is reduced with prolonged culture duration, which hinders the adoption of biotechnological applications for genetic conservation. Germline transmission of PGCs also declines with extended culture duration [45]. Therefore, many studies have been conducted to overcome this limitation. In one study, scientists developed a robust method for the isolation and proliferation of PGCs from the gonads of the embryo [4]. PGCs grew 1 × 10^6^ in 2 weeks in more than 90% of the isolates. Substantial migration of PGCs to the embryonic gonads took place after transplantation of the cells into chicken embryos, and donor-developed offspring were acquired, indicating the ability of PGCs to sustain germline transmission.

#### 1.2.2. Identification of PGCs

Chicken primordial germ cells have peculiar shapes and positions. They possess a spherical nucleus with a separate nucleolus inside and the prevalence of multiple lipid particles in the cytoplasm. Due to their distinct characteristics and large morphology, PGCs circulating through the bloodstream towards the genital ridge are easily distinguished from erythrocytes. The average diameter of PGCs is 10–20 µm. The cytoplasm of PGCs is heavily glycogenotic. They were discovered through periodic acid–Schiff (PAS) reaction staining [3]. PAS staining detects chicken PGCs after 18–19 h of incubation. PGCs of Japanese quails, in contrast, have very little cytoplasmic glycogen and cannot be seen by PAS staining. Even though the precise role of cytoplasmic glycogen in avian PGCs is still unknown, this variation in PAS reactivity has been used to determine the origin of PGCs in specific germline chimeras between the Japanese quail and chicken.

Monoclonal antibodies produced against cell surface antigens—including stage-specific embryonic antigen-1 (SSEA-1), embryonic mouse antigen-1 (EMA-1), SSEA-4, and SSEA-3—have also been used to identify chicken PGCs immunohistochemically. Six-day-old chick embryo gonad extracts were used to create the monoclonal antibody 2C9, which can detect female and male PGCs up to days 8 and 6, respectively. Usually, these markers do not precisely trace the origin of chicken PGCs. Additionally, during development, these markers do not always detect complete PGCs. In contrast, the proportion of VASA-positive PGCs and EMA-1 increases significantly to 89% at stages 20–22 (3–3.5 days of incubation). For instance, only 24% of stage 8 (26–29 h of incubation) VASA-positive PGCs inside the germinal crescent area were found by EMA-1. Stage-specific embryonic antigen-1 (SSEA-1) has been extensively utilized as a suitable marker to recognize and isolate the PGCs of chicken embryos. SSEA-1 is expressed in a variety of different cells and organs in the embryos of chickens; as a result, it is insufficient to observe PGCs at the 5–7 h stage (19–26 h of cultivation, i.e., incubation). Furthermore, not all PGCs express SSEA-1; only a portion of VASA-positive PGCs between stages 8 and 19 do so, albeit at a rising rate [24,28,37,42]. These findings illustrate the phenomenon that PGCs develop via maternally inherited components before the epiblast. However, further studies are required to determine the constituents of the germplasm and the precise role in chalking out germ cell differentiation in avian species.

### 1.3. Research Progress on In Vitro Culture Line Formation and Differentiation in PGCs

#### Propagation of Cultured Primordial Germ Cells In Vitro

Many studies have been performed to determine avian PGCs in vitro. The maintenance and propagation of PGCs were the main objectives of these studies. Previous studies focused on the expansion and number of PGCs for genetic preservation. Recently, embryonic germ cells (EGCs) and embryonic stem cells (ESCs) have been successfully separated from chickens.

Embryonic stem cells are mesodermal stem cells that are obtained from PGCs. EGCs renew themselves through the expansion of an undifferentiated state. Hence, it is essential to study these cells, as they play a vital role in transgenesis and genetic modification in chickens, and they are a significant part of the epigenesis and migration of PGCs. Additionally, the study of stem cells and germ cells helps to produce chimeric animals containing transmission through the germline. Recently, transplantation research has also developed various models that aim to define their extensive utilization to treat a wide range of conditions and diseases in humans, including not only diabetic issues but also neurological and urological disorders [46].

***Embryonic Stem Cells (ESCs)***: In vitro culture of chicken ESCs was first performed in the late 1990s. Later, in a transplantation assay, it was shown that chicken embryonic stem cells isolated from culture after 1–3 passages were capable of producing high-level somatic chimeras. Two somatic germline chimeras that received seven days of chicken ES cell growth demonstrated germline contributions. However, scientists have also found that germline competency becomes extremely low and is practically destroyed when cells are grown for a long time. Transferring chicken embryonic stem cells into the bloodstreams of embryos at stages 12 to 17 may improve the migration of germinal competent cells due to the quick movement of the last cells across the bloodstream to reach the gonads. If long-term cultured chicken ES cells can pass on to the next generation, this culture technique will be an invaluable tool for biotechnology, including modification [47].

***Embryonic Germ Cells (EGCs)*:** EGCs have been developed from PGCs in chickens, which are obtained from embryos at day 5.5 of growth. In order to establish chicken EG cells, a two-step method is used. In the first phase, PGCs form colonies immediately on cells called stroma that are obtained from the somatic cells in the plated gonadal ridge. The second phase, in which multiplying PGCs are coated with chicken embryonic fibroblasts that have not been mitotically disabled, results in a further homogeneous culture. Chicken embryonic germ cells can effectively form embryoid bodies and develop into various cell types when cultivated in suspension. When infused into the subgerminal cavity of stage X embryos, chicken embryonic germ cells can grow in vivo into an assortment of organs. Additionally, after being relocated into the bloodstream of stage 17 embryos, chicken embryonic germ cells obtained from culture after one or two passages grow into functional gametes and generate sustainable offspring. The efficacy of such EG cells for genetic administration is violently hindered by the quick loss of this skillful germline ability in long-term in vitro cultures [48,49].

***Long-Term Cultivation of PGCs***: Previously, a novel method for keeping chicken PGCs committed to the germ cell lineage during long-term culture was discovered. Embryonic blood from stage 14 to stage 17 embryos surrounding PGCs is planted on buffalo rat liver (BRL) feeder cells or STO in knockout Dulbecco’s modified Eagle’s medium accustomed to BRL cells, which are established to produce IGF-1, SCF, and LIF, and include human recombinant and bFGF SCF.

When germline-specific indicators such as DAZL and VASA are expressed, these cells have a ring-shaped appearance, do not stick to the substrate, and proliferate. At stages 13–15 in embryos, these cells are infused into their bloodstream; later, these cells communicate to the next age group at rates ranging from 0.1% to 86%. However, even when injected into stage X embryos, they do not participate in the formation of somatic tissues. It is fascinating to note that when cultured PGCs are placed in a medium bereft of SCF, bFGF, and chicken serum, they expand into follower cells with EG cell traits. Even though they are now proficient in contributing to somatic tissues, these EG-like cells do not contribute to the germline. Furthermore, standard methods are used to cryopreserve cultured chicken PGCs successfully. Therefore, this chicken PGC culture technique can be an effective instrument for biotechnological attempts, including genetic modification and conservation. However, there are variations between male and female PGCs in terms of how effectively their cultures may be derived, with male PGCs having an apparent advantage. Female chimeras transmit female PGCs to the following generation at frequencies of 1–69% in culture for 47 and 66 days, even though these cell lines cannot be retained for longer than 109 and 77 days, respectively [44,45].

### 1.4. Chicken Cell Line Formation

Gametes, produced by germ cells, can vertically transmit genetic material from parents to offspring, acting as a link between generations. According to preformation theory and epigenesis, germ cells either integrate maternal determinants or receive inductive signals from nearby somatic cells during bilateral development.

#### 1.4.1. Epigenesis of PGCs

Epigenetic crescendos are essential for embryonic growth and PGCs. Many studies are still required in avian species to determine the nature of epigenetic reprogramming. However, in this section, a few gathered studies are mentioned and described briefly. The epigenetic profile of chicken PGCs has been reported to contain histone changes, post-transcriptional control by short RNAs, and DNA methylation. DNA methylation itself is a whole process and field of study. Through methylation, mammalian PGCs induce monoallelic expression of anchoring genes while also inactivating one of the two X chromosomes, suppressing gene expression, and maintaining the inactivated retrotransposons [42]. Male and female PGCs have differentially methylated areas, which are also seen in implantation and in mammals’ X-linked homologous areas, indicating that the mechanism governing epigenetic regulation is evolutionarily preserved between birds and mammals [50]. On the other hand, post-translational histone changes and DNA methylation also control the cellular fate of PGCs. In addition, during the development of heterochromatin, chicken PGCs show trimethylation of histone H3 on lysine 9 (H3K9me3) [41]. Regarding post-transcriptional control, mouse, zebrafish, and fly PGCs and germline development depend on short RNAs, such as microRNAs (miRNAs) and PIWI-interacting RNAs (piRNAs). Tiny RNAs are understood to play essential roles in preserving the integrity of germ cells in chickens. Using microarray research, Lee et al. identified chicken PGC-specific miRNAs. In particular, miR-181-3p plays a variety of roles in chicken PGCs, such as preventing PGCs’ progression through the meiotic stage by lowering somatic maturation and nuclear receptor subfamily 6 member 1 (NR6A1) and group A expression. Recently, various piRNAs that are specific to germ cells have been discovered in chicken PGCs. Depending on their genetic sources, these piRNAs can be categorized as being formed from repetitive element sequences of protein-coding genes. These piRNAs and piRNA pathway genes are necessary for chicken PGCs’ genomic integrity, just like in other species. In conclusion, studies on epigenetics in avian germ cells are still in their early stages. Next-generation sequencing technology has recently been used in investigations to clarify the epigenetic roles of avian PGCs [15,42,51].

#### 1.4.2. Germ Cell Gametogenesis in Chicken

Gametogenesis is the process whereby bipotential primordial germ cells grow into specialized generative cells called gametes (oocytes/sperm). In contrast to other species, PGCs in avian embryos reach the future gonadal region via the blood circulation. Because avian PGCs are so easily accessible in the early stages of development, there is a chance to harvest and transplant PGCs.

During the initial germ cell identification stage in chickens, the primordial germ cells (PGCs)—consisting of considerably large, extensive, spherical nuclei and periodic acid–Schiff-coupled refractive cytoplasmic lipids—are helpful in easy identification [44]. Later, germline cell generation in chickens occurs. Creating gametes during gamete formation passes the genetic information from parent to offspring, where germ cells function as a connection between generations. The preformation hypothesis suggests that maternal determinants are incorporated into germ cells during bilateral development. In contrast, the alleged preformation model proposes that synthetic signals from neighboring somatic cells are used to specify germ cells [52]. The preformation model serves as the foundation for chicken germline specification. The next stage can be seen as the migration stage, where the migration of PGCs in chickens occurs [53,54].

In gametogenesis in chickens at stage 39, after 13 days of incubation, genetically male PGCs develop into spermatogonia in the testes before going into a resting phase. At 10 weeks after hatching, the spermatogonia resume cell division and subsequently embark on a differentiation process. Male germ cells do not start proliferating until they reach sexual maturity, at which point spermatogenesis starts and mature sperm forms. However, genetically female PGCs begin their transformation into primary oocytes at stage 34 in the left ovary, corresponding to the eighth incubation day. The first meiosis begins in the left ovary at stage 39 and ends immediately after hatching at the diplotene stage, also known as the fourth stage of the prophase of meiosis. At stage 43, the number of germ cells rises. The number of germ cells thereafter decreases to two-thirds of its peak level one day after hatching. A high rate of apoptosis, also known as programmed cell death in germ cells, is the cause of the reduction in germ cells before hatching [55]. The importance of gametogenesis needs no description. One of the main challenges in regenerative medicine is modifying biological activities, such as division and organization in culture. Stem cell technology plays a vital part in this process. Because they can differentiate into gametes, pluripotent stem cells and spermatogonia stem cells are essential for replicating germ cell development in vitro. In vitro gametogenesis and the reconstruction of germ cell development can offer an experimental framework for a deeper comprehension of germ cell development, as well as a different source of gametes for reproduction, with the potential to treat infertility. We highlight recent developments in chicken genome studies in Table 2.

## 2. The Application of PGCs

### 2.1. Cryopreservation of PGCs—A Frozen Storage Technique

Over the past few decades, chicken breeds have faced various genetic changes; the evolution in their behavior, adaptation of traits, reproduction, and morphology are the main changes that they have faced during the past few decades. All of these things make their local varieties precious in terms of cultural heritage. The chicken is also considered to be the most numerous domestic animal. Seventy years ago, cryopreservation techniques were introduced to preserve specific specimens (i.e., semen and eggs). The process involves the collection of semen or eggs, evaluating them by dilution, cooling, equilibration, adding CPA, and then freezing in liquid nitrogen. Whenever the samples were subjected to usage again, a process of thawing was followed. Although cryopreservation is an excellent technique, it still contains many drawbacks. The significant steps of cryopreservation may lead to irregular cell structure, solution effects, membrane protein and lipid recognition, and oxidative stress.

Cryopreservation is a method for preserving cells and tissues by using low temperatures. The cryopreservation of PGCs is performed via slow freezing or vitrification. Many studies have been conducted on the improvement of this technique. So far, we know that PGCs are valuable for cell-based genetic engineering, genetic modification and preservation, and germplasm expansion. Certainly, PGCs can be developed in culture as mentioned above and cryopreserved with alterations in their biological properties. As PGCs are vital for chicken transgenesis, their preservation is also essential.

Consequently, PGCs appear to be the best candidates for cryopreservation. Two main methods are used for the cryopreservation of living cells and tissues: vitrification, and slow freezing. Slow freezing of PGCs is usually performed using serum-containing media with the addition of DMSO and ethylene glycol. Today, commercially prepared premixed media are also available, which can be used successfully. At the same time, less data and research are available for the cryopreservation of PGCs using the vitrification technique. However, vitrification is considered to be more efficient than slow freezing—especially for the cryopreservation of oocytes, stem cells, and embryos—in terms of cell survival and stability [60].

The significance of cryopreservation is mentioned briefly in this section. In short, sustainability and biosecurity in poultry production are challenging compared to other livestock species. This is because embryo preservation is not very feasible for egg-laying species. Therefore, cryopreservation of PGCs is a potential solution. This can help to produce sufficient independent lines of male and female PGCs that would be appropriate to rebuild a chicken breed. Cryopreservation of PGCs is a major step in biobanking. By using cryopreservation, we can conserve beneficial traits to meet chickens’ current and future production needs.

### 2.2. Research Progress of Primordial Germ Cells (PGCs)—A Cell-Based Gene Transfer Method

In avian species, oocytes are the main precursor cells, commonly known as primordial germ cells. They are referred to as the inherited mode, as well as the preformation mode. There are a lot of gene transfer methods. Compared to other gene transfer methods, the cell-based gene transfer method is the most attractive. The cell-based approach to gene insertion is appealing because it enables the generation of transgenes with specific genome modifications and allows for the examination of transgenes’ expression and integration into the genome before cell implantation into the embryo. Obtaining cultures of cells that can reach the germline (i.e., primordial germ cells), cultivating them, transfecting them with DNA constructs, and implanting the transfected cells into an embryo constitute the initial stages in cell-based transgenic manufacturing. The G0 progeny are then mated to create G1 hens, which have the transgene implanted throughout their entire body. This technical framework combines the efforts of three technologies: the ability to cultivate germline-capable cells, the creation of acceptable DNA constructs, and the production of germline chimeras. The chicken germline chimera was first created by researchers in the lab [58]. The creation of avian germline chimeras has since become a routine process, and techniques for cultivating chicken germ cells, embryonic stem cells, and DNA vectors have all been established. Therefore, while in vitro germline alteration may be used to produce transgenic birds, it has not yet proven successful.

**Figure 5:**
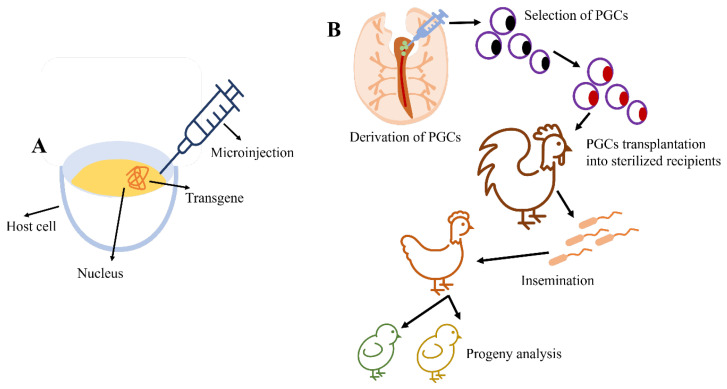
(**A**) Illustration of the DNA microinjection technique; (**B**) transgenesis in chickens using PGCs (modified from Mozdziak and Petitte [29]).

**Figure 6:**
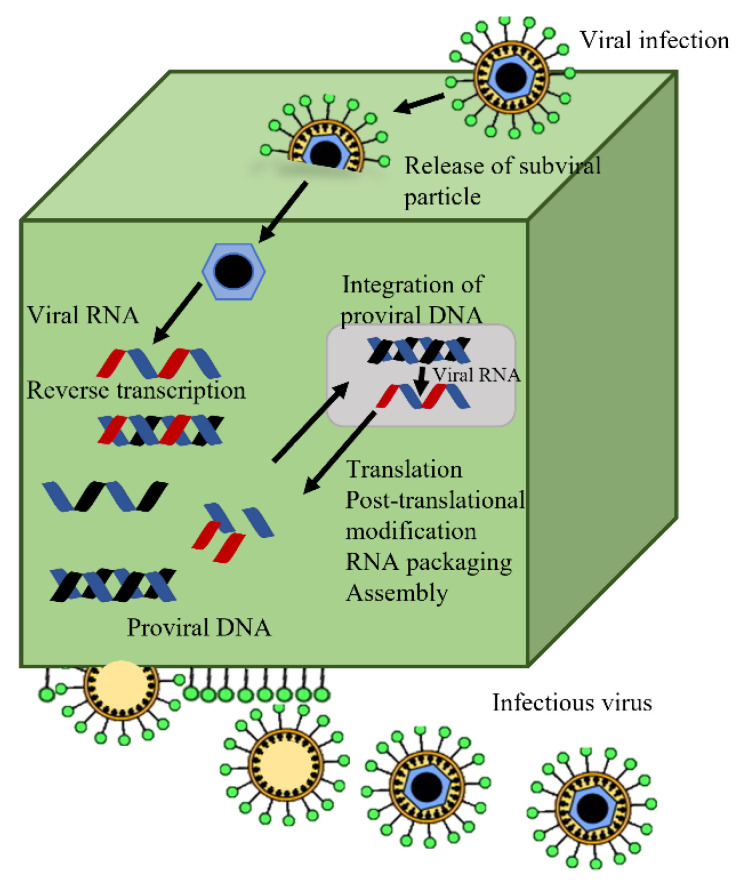
The appeal of retroviral vectors for transgenesis is seen in the usual retrovirus life cycle. Viral RNA is reverse-transcribed into proviral DNA during viral infection, and this proviral DNA is further incorporated into the host genome (modified from Mozdziak and Petitte [29]).

Despite this, all of the necessary techniques are accessible. Finally, there have been some recent developments in sperm-mediated gene transfer. In a nutshell, sperm and a DNA construct are combined, and the combination is utilized to inseminate a fertile female. The sperm that efficiently fertilizes the egg carries the transgene of interest, which is then present in the resulting embryo and hatchlings. Transgenic mammal production has recently been claimed to be reproducible using sperm-mediated transfer. However, the successful integration of a transgene of interest into the avian germline has not yet been demonstrated using sperm-mediated gene transfer. To create transgenic birds, only retroviral and microinjection technology has been used to date [26,61]. Figure 5 demonstrates the microinjection and transgenesis of PGCs. There are several methods used for the transgenesis of chickens [62]. These methods include (1) DNA microinjections, (2) primordial germ cells, (3) gene transfer using non-viral vectors, and (4) gene transfer using retroviral vectors (Figure 6).

## 3. Future Aspects and Considerations

Future developments in agriculture, biotechnology, and scholarly research can be significantly accelerated by combining germline replacement procedures with the manipulation of SSCs or PGCs and the semi-sterilization of recipient embryos, including culturing, freezing, and genome editing. More in-depth research is required to further our understanding of the differences in biological characteristics and functions between female and male germ cells in chickens. To optimize PGC cultures, further research is required as to the effectiveness of culture.

By following a PGC in vitro amplification protocol before cryopreservation, we can avoid the dominant PGC line. Future attempts to construct an outbred flock from cryopreserved cells will require a multiplexing phase to improve the genetic variety of the progeny derived from each surrogate host.

In this research, the expression of the chicken homolog of the deleted in azoospermia-like (c*DAZL*) gene during the specification of germ cells was examined to learn more about the genesis of primordial germ cells (PGCs) and the dynamics of the germplasm. According to the findings, chicken PGCs are transcriptionally active during specification as early as EGK stage VI, which may be the reason for the scattered expression pattern of *DAZL* mRNA and protein. Therefore, relevant gene expression in these early stages should be examined in subsequent research to address the mechanisms of transcriptional alterations in chicken PGCs [25].

## 4. Conclusions

In this review, we discussed the previous studies and research pertaining to chicken PGCs. For many years, various kinds of research have been conducted to understand the origin, development, function, and propagation of germ cells in avian species. These individual cells hold the entirety of the host’s genetic data. With the difficulties in numerous types of avian research, we came to obtain general knowledge on germ cells of avian species, including their specification, origin, identification, migration, epigenesis, propagation, and gametogenesis. However, many factors are still unknown and need deep study and further illustrations, including the cell origin and regulatory mechanisms for the development of germ cells, as well as epigenetic factors. New pioneering technologies for isolation, genome editing, and culture systems of germ cells are likely to provide further awareness of the origin and the evolutionary fate of PGCs.

## Figures and Tables

**Table 1:** Early studies in the development of PGCs.

Year of Study	Name of Study	References
**1870**	Eirstock und Ei. Eine Beitrag zur Anatomie und Entwicklungsgeschichte der Sexualorgane.	[20]
**1880**	Zur Differenzierung des Geschlechts im Tierreich	[32]
**1914**	Origin and early history of the primordial germ-cells in the chick	[33]
**1928**	The history of germ cells in the domestic fowl	[34]
**1996**	Origin of primordial germ cells in the prestreak chick embryos	[35]
**2006, 2008**	Analysis of chicken primordial germ cells Production of chick germline chimeras from florescence-activated cell sorted gonocytes	[36,37]
**2006**	In vivo migration: a germ cell perspective Blimp1 associates with Prmt5 and directs histone arginine methylation in mouse germ cells	[38,39]

**Table 2:** The most recent chicken genome-editing studies using PGCs.

Species	Mediator	Main Research	Purpose	References
Chicken	PGCs	A technical platform mediated by Cas9n was applied to the PGCs, and via the germline transmission system, the myostatin-knockout chickens were generated.	This mutation helps to achieve a hypermuscular phenotype in chickens. This will help promote the growth factor and lead to double-muscle development—a major economic trait in the livestock industry	[56]
Chicken	PGCs	In this study, an amino acid was simply deleted from the gene encoding to infect the chicken cells. The deleted amino acid acts as a receptor for avian leukosis virus subgroup J	The generated mutation is beneficial to produce resistance to avian leukosis virus subgroup J. This virus is an important pathogen in the poultry industry	[57]
Chicken	PGCs	PGC-mediated germline transmission was performed using the CRISPR/Cas9 tool to knockout G0/G1 and switch it by Gene 2	This mutation helps to drastically reduce the abdominal fat in chickens, thereby improving their body fat ratio without altering their other traits	[58]
Chicken	PGCs	GFP was knocked into the z chromosome through the CRISPR/Cas9 tool.	GFP expressing progenies for sexing. Sexing model development was performed; this can help to detect sex during embryogenesis	[59]

## Data Availability

Not applicable.

## References

[1] Himmel L.E., Wilson K.L., Santagostino S.F., Bolon B., Haschek W.M., Rousseaux C.G., Wallig M.A., Bolon B. (2022). Chapter 23—Genetically Engineered Animal Models in Toxicologic Research. Haschek and Rousseaux’s Handbook of Toxicologic Pathology.

[2] Doran T.J., Cooper C.A., Jenkins K.A., Tizard M.L. (2016). Advances in genetic engineering of the avian genome: “Realising the promise”. Transgenic Res..

[3] Tagami T., Miyahara D., Nakamura Y. (2017). Avian Primordial Germ Cells. Adv. Exp. Med. Biol..

[4] Xie L., Lu Z., Chen D., Yang M., Liao Y., Mao W., Mo L., Sun J., Yang W., Xu H. (2019). Derivation of chicken primordial germ cells using an indirect Co-culture system. Theriogenology.

[5] Richardson B.E., Lehmann R. (2010). Mechanisms guiding primordial germ cell migration: Strategies from different organisms. Nat. Rev. Mol. Cell Biol..

[6] Meng L., Wang S., Jiang H., Hua Y., Yin B., Huang X., Man Q., Wang H., Zhu G. (2022). Oct4 dependent chromatin activation is required for chicken primordial germ cell migration. Stem Cell Rev. Rep..

[7] Zuo Q., Jing J., Zhou J., Zhang Y., Wei W., Chen G., Li B. (2022). Dual regulatory actions of LncBMP4 on BMP4 promote chicken primordial germ cell formation. EMBO Rep..

[8] Andersson L., Georges M. (2004). Domestic-animal genomics: Deciphering the genetics of complex traits. Nat. Rev. Genet..

[9] Han J.Y., Park Y.H. (2018). Primordial germ cell-mediated transgenesis and genome editing in birds. J. Anim. Sci. Biotechnol..

[10] Lee H.J., Lee H.C., Han J.Y. (2015). Germline Modification and Engineering in Avian Species. Mol. Cells.

[11] Gordon J.W., Scangos G.A., Plotkin D.J., Barbosa J.A., Ruddle F.H. (1980). Genetic transformation of mouse embryos by microinjection of purified DNA. Proc. Natl. Acad. Sci. USA.

[12] Hammer R.E., Pursel V.G., Rexroad C.E., Wall R.J., Bolt D.J., Ebert K.M., Palmiter R.D., Brinster R.L. (1985). Production of transgenic rabbits, sheep and pigs by microinjection. Nature.

[13] Thomas K.R., Capecchi M.R. (1987). Site-directed mutagenesis by gene targeting in mouse embryo-derived stem cells. Cell.

[14] Han J.Y. (2009). Germ cells and transgenesis in chickens. Comp. Immunol. Microbiol. Infect. Dis..

[15] Lee H.C., Choi H.J., Park T.S., Lee S.I., Kim Y.M., Rengaraj D., Nagai H., Sheng G., Lim J.M., Han J.Y. (2013). Cleavage events and sperm dynamics in chick intrauterine embryos. PLoS ONE.

[16] Herron L.R., Pridans C., Turnbull M.L., Smith N., Lillico S., Sherman A., Gilhooley H.J., Wear M., Kurian D., Papadakos G. (2018). A chicken bioreactor for efficient production of functional cytokines. BMC Biotechnol..

[17] Farzaneh M., Hassani S.-N., Mozdziak P., Baharvand H. (2017). Avian embryos and related cell lines: A convenient platform for recombinant proteins and vaccine production. Biotechnol. J..

[18] Pal A. (2022). Transgenesis and Biopharming. Protocols in Advanced Genomics and Allied Techniques.

[19] Love J., Gribbin C., Mather C., Sang H. (1994). Transgenic birds by DNA microinjection. Bio/Technology.

[20] Eyal-Giladi H., Kochav S. (1976). From cleavage to primitive streak formation: A complementary normal table and a new look at the first stages of the development of the chick. I. General morphology. Dev. Biol..

[21] Zhu L., van de Lavoir M.C., Albanese J., Beenhouwer D.O., Cardarelli P.M., Cuison S., Deng D.F., Deshpande S., Diamond J.H., Green L. (2005). Production of human monoclonal antibody in eggs of chimeric chickens. Nat. Biotechnol..

[22] Capecchi M.R. (2005). Gene targeting in mice: Functional analysis of the mammalian genome for the twenty-first century. Nat. Rev. Genet..

[23] Waldeyer W. (1870). Eierstock und Ei: Ein Beitrag zur Anatomie und Entwicklungsgeschichte des Sexualorgane.

[24] Hamburger V., Hamilton H.L. (1992). A series of normal stages in the development of the chick embryo. Dev. Dyn..

[25] Eyal-Giladi H., Ginsburg M., Farbarov A. (1981). Avian primordial germ cells are of epiblastic origin. J. Embryol. Exp. Morphol..

[26] Tsang T.E., Khoo P.L., Jamieson R.V., Zhou S.X., Ang S.L., Behringer R., Tam P.P. (2001). The allocation and differentiation of mouse primordial germ cells. Int. J. Dev. Biol..

[27] Tsunekawa N., Naito M., Sakai Y., Nishida T., Noce T. (2000). Isolation of chicken vasa homolog gene and tracing the origin of primordial germ cells. Development.

[28] Lee H.C., Choi H.J., Lee H.G., Lim J.M., Ono T., Han J.Y. (2016). DAZL Expression Explains Origin and Central Formation of Primordial Germ Cells in Chickens. Stem Cells Dev..

[29] Mozdziak P.E., Petitte J.N. (2004). Status of transgenic chicken models for developmental biology. Dev. Dyn. Off. Publ. Am. Assoc. Anat..

[30] Ginsburg M., Eyal-Giladi H. (1987). Primordial germ cells of the young chick blastoderm originate from the central zone of the area pellucida irrespective of the embryo-forming process. Development.

[31] Urven L.E., Erickson C.A., Abbott U.K., McCarrey J.R. (1988). Analysis of germ line development in the chick embryo using an anti-mouse EC cell antibody. Development.

[32] Nussubaum M. (1880). Zur Differenzierung des Geschlechts im Tierreich. Arch. Mikrosk. Anat..

[33] Swift C.H. (1914). Origin and Early History of the Primordial Germ-Cells in the Chick.

[34] Goldsmith J.B. (1928). The history of the germ cells in the domestic fowl. J. Morphol..

[35] Karagenç L., Cinnamon Y., Ginsburg M., Petitte J.N. (1996). Origin of primordial germ cells in the prestreak chick embryo. Dev. Genet..

[36] Motono M., Ohashi T., Nishijima K., Iijima S. (2008). Analysis of chicken primordial germ cells. Cytotechnology.

[37] Mozdziak P.E., Wysocki R., Angerman-Stewart J., Pardue S.L., Petitte J.N. (2006). Production of chick germline chimeras from fluorescence-activated cell-sorted gonocytes. Poult. Sci..

[38] Ancelin K., Lange U.C., Hajkova P., Schneider R., Bannister A.J., Kouzarides T., Surani M.A. (2006). Blimp1 associates with Prmt5 and directs histone arginine methylation in mouse germ cells. Nat. Cell Biol..

[39] Kunwar P.S., Siekhaus D.E., Lehmann R. (2006). In vivo migration: A germ cell perspective. Annu. Rev. Cell Dev. Biol..

[40] Glover J., McGrew M. (2012). Primordial Germ Cell Technologies for Avian Germplasm Cryopreservation and Investigating Germ Cell Development. J. Poult. Sci..

[41] Kang K.S., Lee H.C., Kim H.J., Lee H.G., Kim Y.M., Lee H.J., Park Y.H., Yang S.Y., Rengaraj D., Park T.S. (2015). Spatial and temporal action of chicken primordial germ cells during initial migration. Reproduction.

[42] Kim Y.M., Han J.Y. (2018). The early development of germ cells in chicken. Int. J. Dev. Biol..

[43] Molyneaux K.A., Zinszner H., Kunwar P.S., Schaible K., Stebler J., Sunshine M.J., O’Brien W., Raz E., Littman D., Wylie C. (2003). The chemokine SDF1/CXCL12 and its receptor CXCR4 regulate mouse germ cell migration and survival. Development.

[44] Nakamura Y., Usui F., Miyahara D., Mori T., Ono T., Takeda K., Nirasawa K., Kagami H., Tagami T. (2010). Efficient system for preservation and regeneration of genetic resources in chicken: Concurrent storage of primordial germ cells and live animals from early embryos of a rare indigenous fowl (Gifujidori). Reprod. Fertil. Dev..

[45] van de Lavoir M.C., Diamond J.H., Leighton P.A., Mather-Love C., Heyer B.S., Bradshaw R., Kerchner A., Hooi L.T., Gessaro T.M., Swanberg S.E. (2006). Germline transmission of genetically modified primordial germ cells. Nature.

[46] Kerr C.L., Gearhart J.D., Elliott A.M., Donovan P.J. (2006). Embryonic germ cells: When germ cells become stem cells. Semin. Reprod. Med..

[47] Nakamura Y., Kagami H., Tagami T. (2013). Development, differentiation and manipulation of chicken germ cells. Dev. Growth Differ..

[48] Park T.S., Han J.Y. (2000). Derivation and characterization of pluripotent embryonic germ cells in chicken. Mol. Reprod. Dev..

[49] Park T.S., Jeong D.K., Kim J.N., Song G.H., Hong Y.H., Lim J.M., Han J.Y. (2003). Improved germline transmission in chicken chimeras produced by transplantation of gonadal primordial germ cells into recipient embryos. Biol. Reprod..

[50] Jang H.J., Seo H.W., Lee B.R., Yoo M., Womack J.E., Han J.Y. (2013). Gene expression and DNA methylation status of chicken primordial germ cells. Mol. Biotechnol..

[51] Lee S.I., Lee B.R., Hwang Y.S., Lee H.C., Rengaraj D., Song G., Park T.S., Han J.Y. (2011). MicroRNA-mediated posttranscriptional regulation is required for maintaining undifferentiated properties of blastoderm and primordial germ cells in chickens. Proc. Natl. Acad. Sci. USA.

[52] Extavour C.G., Akam M. (2003). Mechanisms of germ cell specification across the metazoans: Epigenesis and preformation. Development.

[53] Nakamura Y., Yamamoto Y., Usui F., Mushika T., Ono T., Setioko A.R., Takeda K., Nirasawa K., Kagami H., Tagami T. (2007). Migration and proliferation of primordial germ cells in the early chicken embryo. Poult. Sci..

[54] Stebler J., Spieler D., Slanchev K., Molyneaux K.A., Richter U., Cojocaru V., Tarabykin V., Wylie C., Kessel M., Raz E. (2004). Primordial germ cell migration in the chick and mouse embryo: The role of the chemokine SDF-1/CXCL12. Dev. Biol..

[55] Ukeshima A. (1996). Germ cell death in the degenerating right ovary of the chick embryo. Zool. Sci..

[56] Kim G.D., Lee J.H., Song S., Kim S.W., Han J.S., Shin S.P., Park B.C., Park T.S. (2020). Generation of myostatin-knockout chickens mediated by D10A-Cas9 nickase. FASEB J. Off. Publ. Fed. Am. Soc. Exp. Biol..

[57] Koslová A., Trefil P., Mucksová J., Reinišová M., Plachý J., Kalina J., Kučerová D., Geryk J., Krchlíková V., Lejčková B. (2020). Precise CRISPR/Cas9 editing of the NHE1 gene renders chickens resistant to the J subgroup of avian leukosis virus. Proc. Natl. Acad. Sci. USA.

[58] Park T.S., Park J., Lee J.H., Park J.W., Park B.C. (2019). Disruption of G(0)/G(1) switch gene 2 (G0S2) reduced abdominal fat deposition and altered fatty acid composition in chicken. FASEB J. Off. Publ. Fed. Am. Soc. Exp. Biol..

[59] Lee H.J., Yoon J.W., Jung K.M., Kim Y.M., Park J.S., Lee K.Y., Park K.J., Hwang Y.S., Park Y.H., Rengaraj D. (2019). Targeted gene insertion into Z chromosome of chicken primordial germ cells for avian sexing model development. FASEB J. Off. Publ. Fed. Am. Soc. Exp. Biol..

[60] Tonus C., Connan D., Waroux O., Vandenhove B., Wayet J., Gillet L., Desmecht D., Antoine N., Ectors F.J., Grobet L. (2017). Cryopreservation of chicken primordial germ cells by vitrification and slow freezing: A comparative study. Theriogenology.

[61] Lavitrano M., Forni M., Bacci M.L., Di Stefano C., Varzi V., Wang H., Seren E. (2003). Sperm mediated gene transfer in pig: Selection of donor boars and optimization of DNA uptake. Mol. Reprod. Dev..

[62] Bahrami S., Amiri-Yekta A., Daneshipour A., Jazayeri S.H., Mozdziak P.E., Sanati M.H., Gourabi H. (2020). Designing A Transgenic Chicken: Applying New Approaches toward A Promising Bioreactor. Cell J..

